# Biomimetic Porous CuO/ZnO S‑Scheme Heterojunction for Highly Efficient Sunlight-Driven Photocatalytic Degradation of Rhodamine B

**DOI:** 10.1021/acsomega.5c06416

**Published:** 2025-09-04

**Authors:** Antony Dasint Lopis, Arshitha Madhusudhan, Bhavana Kulkarni, Sanjeev Maradur, Khoobaram S. Choudhari, Suresh D. Kulkarni

**Affiliations:** † Manipal Institute of Applied Physics, 76793 Manipal Academy of Higher Education, Manipal, Karnataka, 576104, India; ‡ Materials Science & Catalysis Division, 226402 Poornaprajna Institute of Scientific Research (PPISR), Bidalur Post, Devanahalli, Bengaluru, Karnataka, 562164, India

## Abstract

Sunlight-driven photocatalysis is at the forefront of sustainable technologies for the remediation of organic pollutants in industrial wastewater. In this study, we present the innovative synthesis of porous microspheres based on an S-scheme CuO/ZnO composite photocatalyst, inspired by the unique morphology of dried resurrection plants. X-ray diffraction confirmed the formation of highly crystalline CuO and ZnO phases, while UV–vis-NIR spectroscopy revealed exceptional sunlight harvesting capabilities, with optical absorption spanning the solar spectrum and a narrow bandgap of 1.4 eV. Under optimal conditions, the photocatalyst achieved a remarkable 97% degradation of Rhodamine B dye within just 60 min of solar irradiation. Systematic evaluation across varying compositions, pH values, and catalyst loadings highlighted robust photocatalytic performance. Moreover, the composite demonstrated outstanding stability and reusability, retaining high activity over more than five consecutive cycles. This full-solar-spectrum-active, biomimetic photocatalyst offers a promising platform for the efficient, sunlight-driven purification of hazardous organic effluents, with significant implications for environmental sustainability and public health.

## Graphical abstract

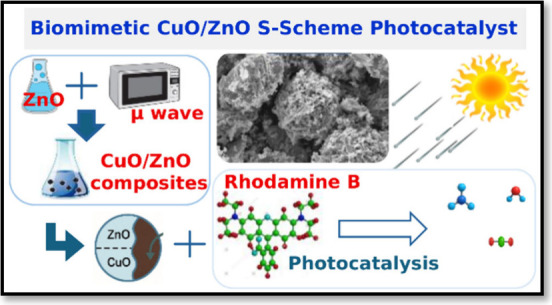

## 1 Introduction

Water scarcity and contamination represent a critical global crisis, with only 2.5% of Earth’s freshwater suitable for human consumption despite water covering two-thirds of the planet.^1^ Rapid industrialization, agricultural runoff from pesticides, fertilizers, and inadequate wastewater treatment have led to widespread contamination of these vital sources, exacerbating global water scarcity.^1,2^ Industrial effluents containing carcinogenic dyes, such as Rhodamine B, are particularly hazardous due to their persistence, toxicity, and bioaccumulation potential.^3^ This pollution directly undermines UN Sustainable Development Goal 6 (clean water and sanitation), necessitating urgent, scalable, and sustainable remediation technologies to protect public health and ecological integrity.

To address the problem of waste dyes entering freshwater bodies, various physical and chemical wastewater treatment methods have been utilized.^2^ Traditional wastewater treatment methods—such as adsorption, ion exchange, filtration, and coagulation—are limited by secondary pollution and material saturation, making them impractical for large-scale dye removal.^4,5^ Chemical approaches, including precipitation and oxidation, are expensive, generate toxic byproducts, and are often ineffective for complex organic pollutants.^2,6,7^

Photocatalysis has emerged as an effective method for decomposing carcinogenic dyes into nontoxic, simpler molecules and minerals. This technique uses semiconductor photocatalysts to generate reactive species that break down complex organic pollutants. Among various photocatalytic approaches, photocatalysis stands out due to the use of low-cost and reusable materials, high degradation efficiency, the ability to achieve complete mineralization without producing harmful byproducts, and the utilization of solar radiation as a sustainable energy source, making it an eco-friendly solution for wastewater treatment.^8^

The effectiveness of photocatalysis depends on the semiconductor’s bandgap.^9^ The narrower the photocatalyst bandgap, the higher the sunlight absorption.^10^ However, a narrower bandgap leads to an increased rate of electron–hole pair recombination, which negatively impacts photocatalysis.^11^ Due to these two conflicting conditions, it is difficult for a single-material photocatalyst to achieve both absorption in the longer wavelength (visible and NIR) region and an extended charge carrier lifetime before recombination.^12^ Therefore, the design of an appropriate heterogeneous photocatalytic material system is a viable solution to this issue. Such a system could take the form of either a core–shell type or a nanocomposite.^13–15^ To achieve this, a low bandgap semiconductor that absorbs the solar spectrum efficiently was combined with a wider bandgap semiconductor to create a heterojunction, thus addressing the recombination problem.^12,16–22^

Copper oxide (CuO), with its narrow bandgap (∼1.4 eV in nanostructured form), is an excellent light harvester but suffers from rapid recombination.^23,24^ Coupling CuO with other semiconductors, such as ZnO, has been shown to improve charge separation and photocatalytic activity.^25^ Recent advances in heterogeneous photocatalysis have explored various Cu–O-based composite materials for the degradation of organic pollutants. For example, Lu et al. demonstrated the photocatalytic decomposition of phenol under visible light using a CuO/TiO_2_ composite as a photocatalyst.^26^ Wang et al. reported the synthesis of a CuO@TiO_2_ nanocomposite photocatalyst and evaluated its performance under visible light for the degradation of azo dyes in industrial wastewater.^27^ Expanding the scope, Nekooie et al. report the synthesis of a novel CuO/TiO_2_/PANI nanocomposite for the visible light-assisted photodegradation of chlorpyrifos.^28^ Bekru et al. report the green synthesis of a CuO–ZnO nanocomposite for the photodegradation of Methylene Blue and the reduction of 4-Nitrophenol.^29^ Mubeen et al. report on tuning the band structure of ZnO/CuO nanocomposites by varying the weight percentage of CuO to enhance photocatalytic activity in the degradation of methylene blue dye.^30^ Recently, Jeevarathinam et al. demonstrated the photodegradation of Rhodamine B dye under UV light using a CuO-ZnO composite.^31^ Dien et al. synthesized CuO nanoplate/ZnO nanoparticle hybrid photocatalysts and reported their photocatalytic performance for the degradation of Methylene Blue under visible light.^32^ Despite these promising results, most reported CuO–ZnO systems have focused on solid- or nanoparticle-based morphologies. There remains a critical opportunity to further enhance photocatalytic activity by engineering porous structures that better mimic natural systems, thereby improving light absorption, surface area, and mass transport, key factors for efficient pollutant removal.

In this work, we present an eco-friendly microwave-assisted hydrothermal synthesis of CuO-ZnO composite microspheres, engineered to mimic the unique morphology of resurrection plants. The resulting porous microspheres (1–3 μm in diameter, surface area ∼ 5.35 m^2^/g) were thoroughly characterized using advanced techniques, including XRD, FESEM-EDS, BET, and UV/vis/NIR spectrophotometry. The photocatalytic performance of the composites was systematically evaluated across various CuO/ZnO ratios, pH conditions, and catalyst loadings with a particular focus on the degradation of Rhodamine B under sunlight. The generation and role of reactive oxygen species were elucidated through scavenging and confirmatory experiments. Our findings demonstrate that this innovative photocatalyst not only exhibits outstanding efficiency and stability but also offers a promising, sustainable solution for the removal of hazardous organic pollutants from industrial wastewater.

## 2 Materials and Methods

### 2.1 Chemicals

Copper­(II) chloride dihydrate (CuCl_2_·2H_2_O, >99%, Merck), Ammonia solution (NH_4_OH, 25%, Emplura), and Deionized (DI) water (18 MΩ). Zinc nitrate hexahydrate (Zn­(NO_3_)_2_·6H_2_O, >96%, Merck), Ethylene glycol (99%, Emplura), Sodium hydroxide (NaOH, Merck), and Deionized (DI) water (18 MΩ).

### 2.2 Synthesis of Copper Oxide Microstructures

To synthesize copper oxide microstructures, 12.57 mM of CuCl_2_·2H_2_O was dissolved in 40 mL of DI water. 3.84 mL of NH_4_OH was added to this solution drop by drop, under continuous stirring for 1 h. Following that, the solution is transferred to an 80 mL quartz vial and microwaved at 160 °C for 20 min in a microwave synthesizer. The synthesized CuO microstructures were separated by centrifugation, washed multiple times with water and ethanol, and dried at 85 °C for 14 h.

### 2.3 Synthesis of Resurrection Plant-Like CuO/ZnO Composite Microspheres

To synthesize resurrection plant-like CuO/ZnO composite microspheres, 1.23 mM of Zn­(NO_3_)_2_·6H_2_O is added to a solution mixture of 15 mL of DI water and 45 mL of ethylene glycol and sonicated for 3 min. The desired amount of synthesized CuO microstructures (Table 1) is added to the above solution and sonicated. Then, the dispersion was transferred into a round-bottom flask and placed in the domestic microwave oven.^33,34^ Simultaneously, 2.44 mM NaOH was dissolved in a mixture of 10 mL of DI water and 30 mL of ethylene glycol. Once the round-bottom flask is placed inside the microwave and its contents start to boil, the NaOH solution is added drop by drop through the condenser.^3^ The total time of microwave heating was restricted to 30 min, and after that, the slurry was cooled to room temperature. The synthesized CuO-ZnO composite was centrifuged, washed, and dried at 85 °C for 14 h. The synthesis procedure is depicted in Scheme 1, outlining the key steps involved in forming the CuO-ZnO composite.

**Scheme 1:**
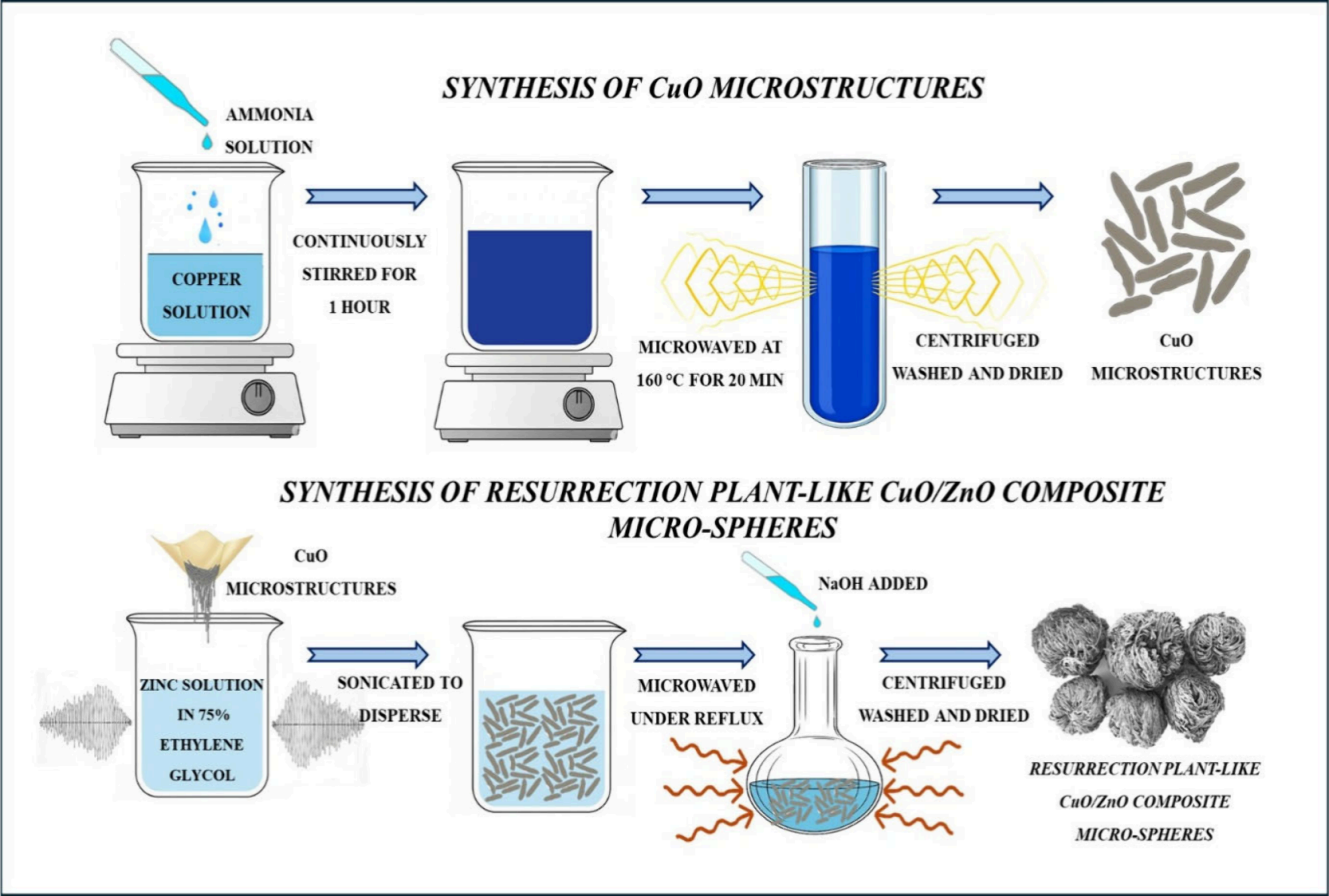
Schematic Illustration of the Synthesis Route for CuO-ZnO Composite Via Co-Precipitation and Calcination

**Table 1:** Experimental Parameters Used for the Preparation of Photocatalysts with Different Compositions

	Amount of CuO added		
Sample Code	**in m moles**	**in mg**	ZnO:CuO
C1	0.82	65	1.5:1
C2	0.62	50	2:1
C3	0.41	33	3:1
C4	0.35	28	3.5:1

### 2.4 Characterization

An X-ray diffractometer (Rigaku Ultima IV) operating at a scanning rate of 2°/min was used to assess the crystallinity of the produced CuO core and CuO-ZnO microstructures in the 2θ range of 20° to 80°. Morphological and elemental characterization was performed using a field emission scanning electron microscope (FESEM, ZEISS ULTRA-55) equipped with an EDS. Before imaging, the materials were sputtered with gold and palladium. The specific surface area, pore volume, and pore size of the nanoparticles were determined from nitrogen sorption measurement using a Surface area analyzer (Belsorb Mini (II), BEL Japan). The isotherms were measured at 77 K after degassing samples below 10^–2^ kPa at 120 °C for 4 h. The materials’ diffuse reflectance spectra were obtained using a PerkinElmer Lambda 750 UV/vis/NIR spectrophotometer. A fluorescence spectrometer fitted with a 150 W xenon lamp and a high-speed chopper (Jasco FP8300) was used to acquire photoluminescence spectra. The absorption spectra of the aliquots were collected using a UV–vis Spectrophotometer (V-650, JASCO, U.K.).

### 2.5 Photocatalytic Activity

A total of 20 mg of the synthesized nanoparticles was added to 50 mL of a Rhodamine B solution. The mixture was stirred in the dark for 30 min to achieve sorption equilibrium. Following equilibrium, the dispersion was exposed to natural sunlight. At predetermined time intervals, approximately 2 mL aliquots were collected and centrifuged to remove the photocatalyst. The concentration of Rhodamine B in the supernatant was then measured by using a single-beam visible spectrometer at 520 nm to follow the degradation throughout the experimental process.

## 3 Results and Discussions

### 3.1 Structural Analysis – X-ray Diffraction (XRD) Analysis

The XRD data shows that the copper oxide has diffraction peaks at 32.68, 35.60, 38.6, 49.06, 53.76, 58.66, and 61.86°, which correspond to the monoclinic structure of copper oxide (JCPDS 48-1548) as shown in Figure 1.^35^

**Figure 1:**
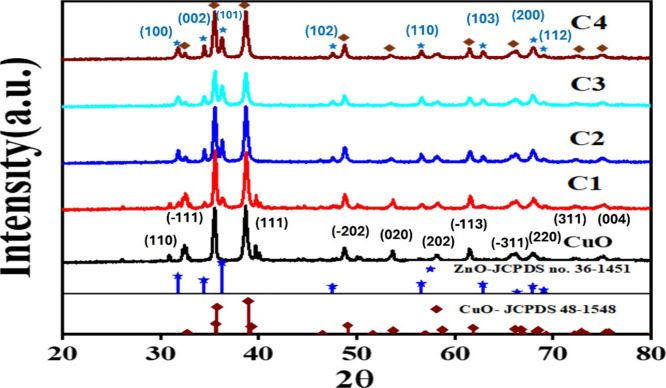
X-ray diffraction (XRD) pattern confirming the formation and high crystallinity of synthesized CuO, C1, C2, C3, and C4 samples.

The crystallite size (d) of the CuO was determined to be 24 nm using Debye–Scherrer’s equation given by, $d=\frac{0.9\lambda }{\beta \cos\theta }$ ; where λ = wavelength of X-ray, β = fwhm of the peak, & θ = peak position (here 2θ = 38.6°). The XRD patterns (Figure 1) of samples C1–C4 showed extra peaks apart from CuO at 31.8, 34.4, 36.2, 47.4, 63.1, and 72.4°, which correspond to the Wurtzite structure of ZnO.^35,36^ This confirmed the formation of crystalline CuO/ZnO composites.

### 3.2 Surface Morphology by FE-SEM

FE-SEM images (Figure 2a,b) revealed structures with dimensions of approximately 1–2 μm in length, 200 nm in width, and 100 nm in thickness. The EDS spectra confirmed the presence of Cu and O, with respective compositions of 43% and 57% (Figure 2c). No additional peaks were observed, except for carbon, which was used for sampling. The slightly higher oxygen content may be attributed to surface-adsorbed oxygen species, such as moisture and hydroxides. Elemental mapping (Figure 2d) demonstrated a uniform distribution of Cu and O throughout the structures.

**Figure 2:**
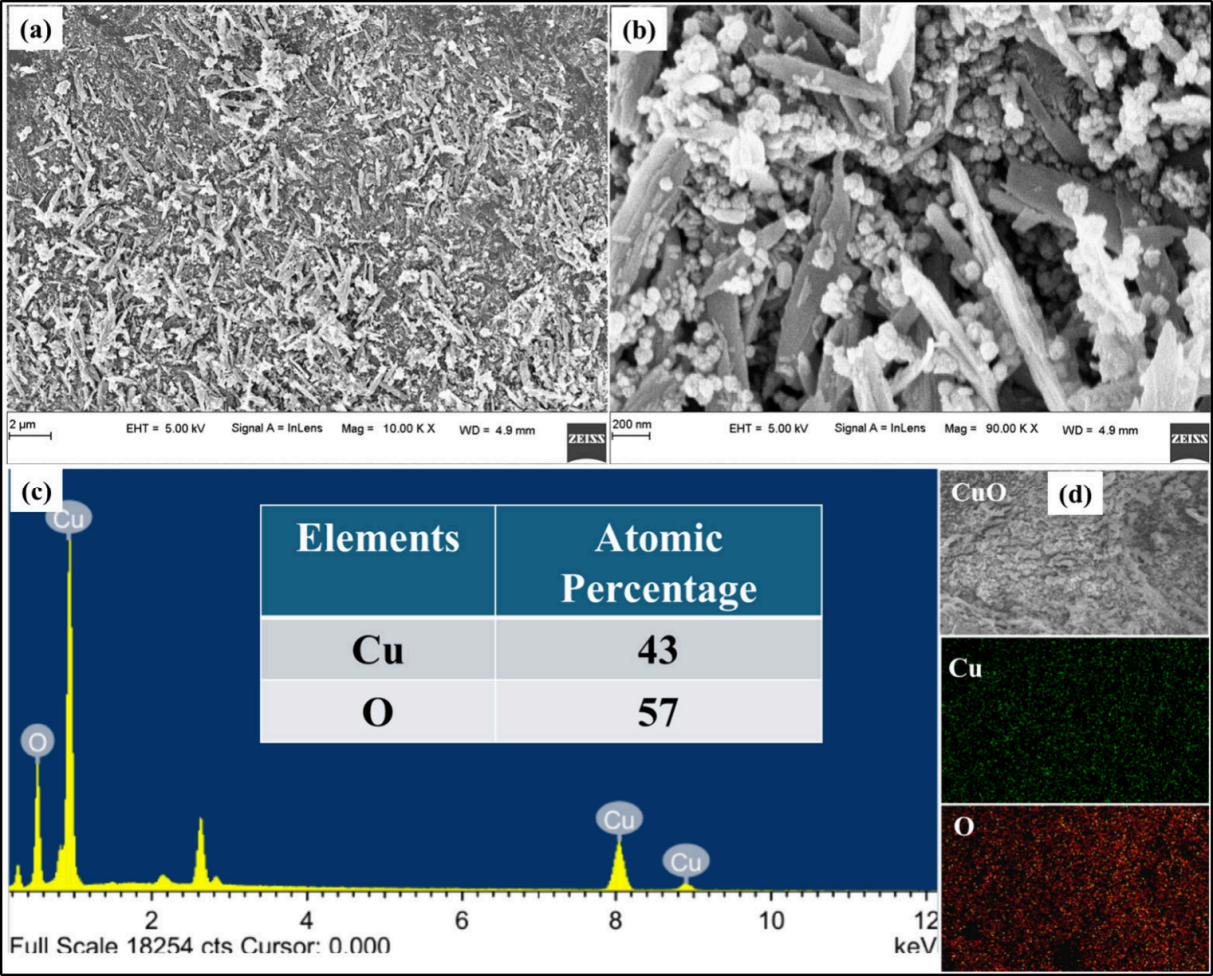
(a, b) Field emission scanning electron microscopy (FESEM) images of CuO microstructures, revealing their size and morphology; (c) Energy dispersive X-ray spectroscopy (EDS) spectrum with inset table showing elemental composition; (d) Elemental mapping illustrating the uniform distribution of Cu and O.

Figure 3a displays microspheres approximately 3 μm in diameter with CuO-like structures embedded within the spheres. These spheres appear to be composed of CuO structures and ZnO nanoparticles (Figure 3b). EDS analysis (Figure 3c) confirmed the presence of Cu, Zn, and O, with respective compositions of 23%, 19%, and 58%. Elemental mapping (Figure 3d) demonstrated the uniform distribution of these elements throughout the sample, proving the formation of a composite.

**Figure 3:**
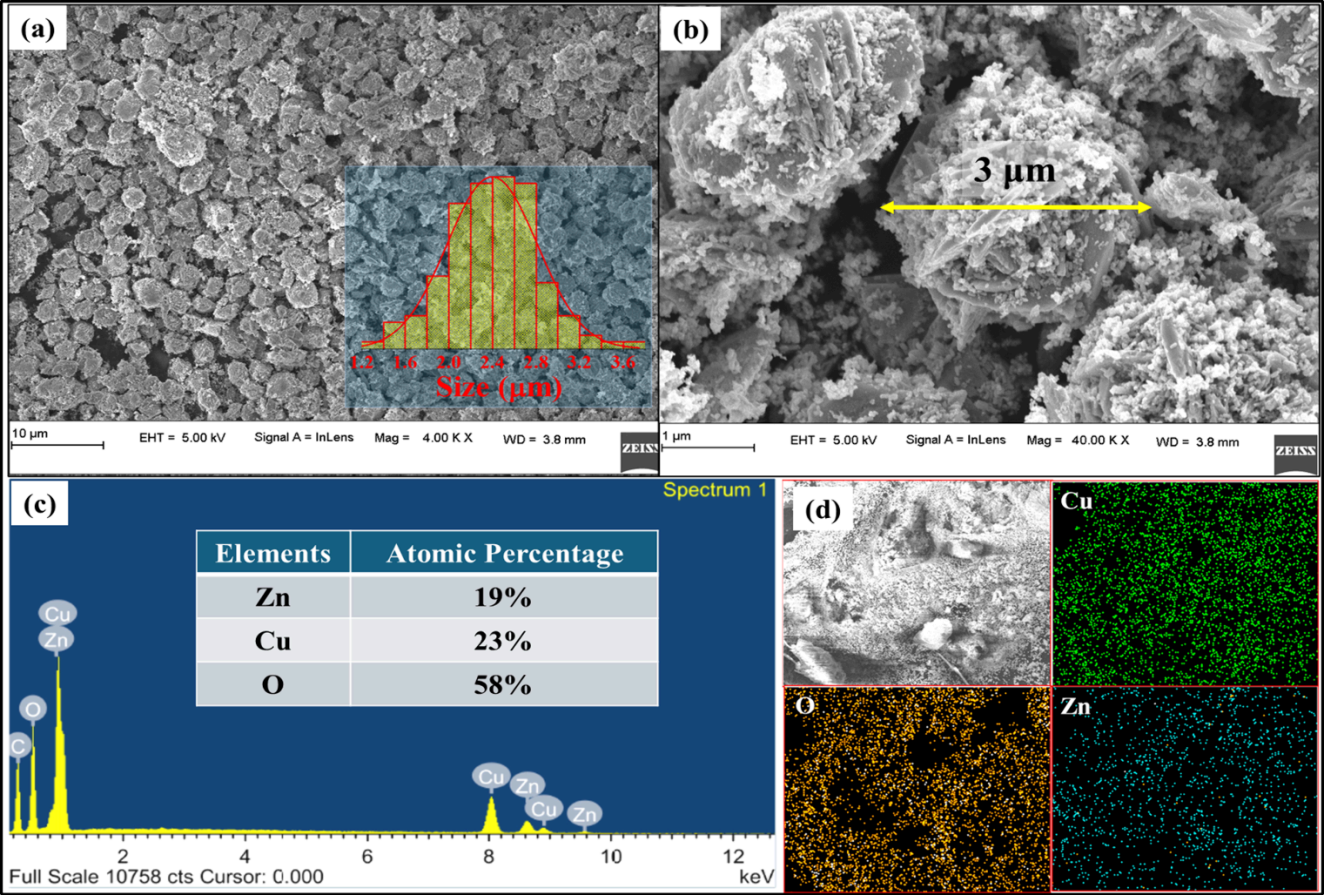
(a) FESEM image of C2 composites with the size histogram, (b) a magnified electron microscopic image, (c) EDS spectrum, and (d) Elemental mapping of C2 composites displaying the uniform distribution of the elements

### 3.3 Optical Characterization

As illustrated in Figure 4a, both CuO and CuO-ZnO showed strong absorption, covering an entire visible region of sunlight and extending to near-infrared. The indirect bandgap of these materials was determined by Kubelka–Munk plots. As shown in Figure 4b, the indirect bandgap of CuO was determined to be 1.4 eV, and it remained unaltered after forming the CuO-ZnO composite. The observed bandgap was consistent with the reports available for CuO particles in these sizes.^37,38^

**Figure 4:**
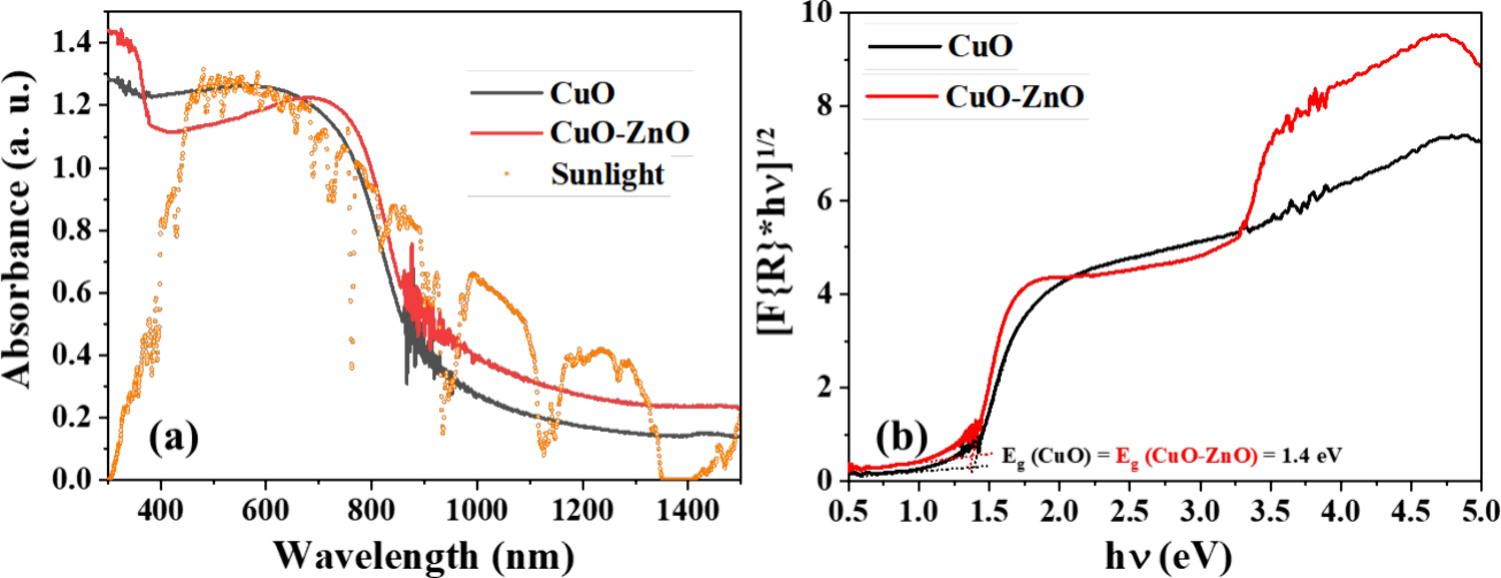
(a) UV–vis-NIR absorbance spectra of CuO and CuO-ZnO (C2) composites, demonstrating their broad solar light absorption; (b) Kubelka–Munk plots used to estimate the indirect bandgaps of CuO and CuO-ZnO (C2), confirming the retention of a narrow bandgap after composite formation.

### 3.4 Surface Area Analysis

The catalytic activity of the particles depends on the active sites available on the catalyst. The larger the surface area of the photocatalyst, the better the degradation kinetics of the pollutants. The CuO-ZnO nanoparticles exhibit a type IVa N_2_ isotherm according to the latest IUPAC report, with a mild hysteresis loop, indicating the presence of some mesopores, as shown in Figure 5a. The surface area of CuO particles was determined to be 1.41 m^2^/g, and it increases upon the formation of the CuO-ZnO composite (Table 2). As indicated by the BJH plots (inset of Figure 5a,b), the volume of the pores increased after the composite formation. The observed values were 0.0074 cm^3^ g^–1^ and 0.0377 cm^3^ g^–1^, respectively, for CuO and CuO-ZnO. The drastic increase in the pore volume led to a higher surface area, even though the size of the particles was about 3 μM as observed by the FESEM.

**Figure 5:**
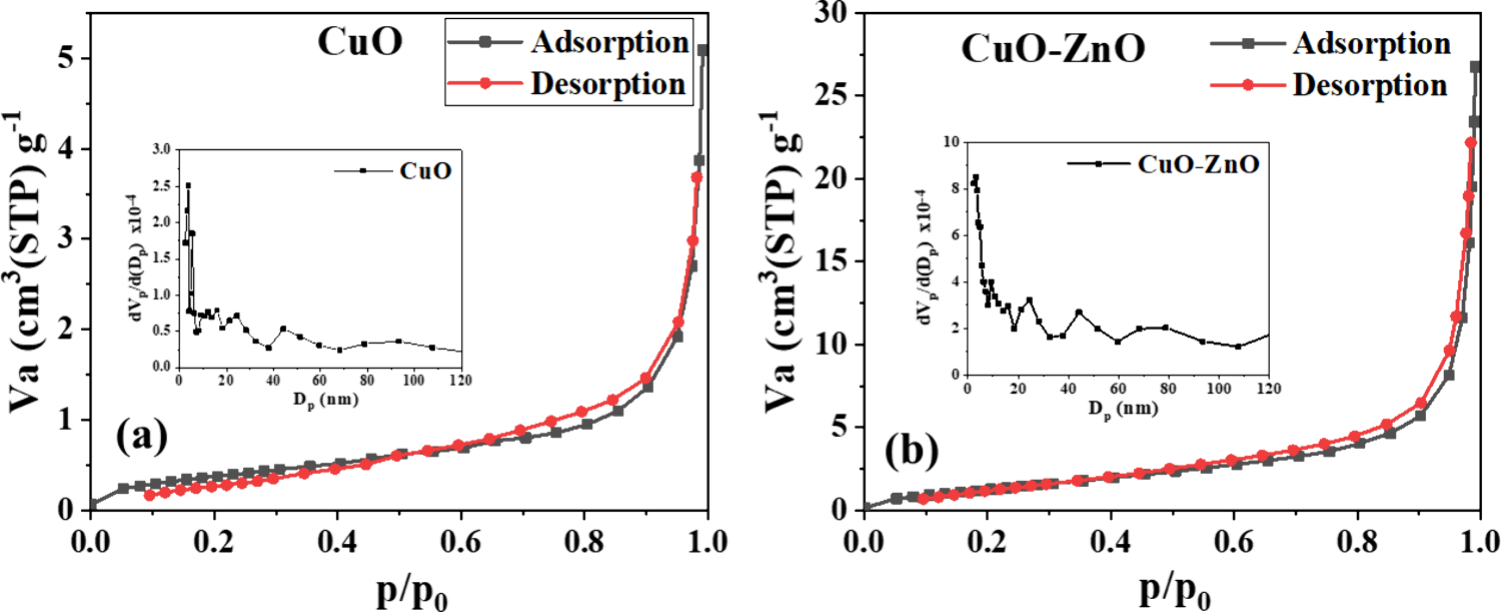
Nitrogen adsorption–desorption isotherms of (a) CuO and (b) CuO-ZnO (C2) composites; insets show corresponding BJH pore size distribution plots.^21^

**Table 2:** BET Surface Area, Pore Volume, and Pore Size of CuO and CuO-ZnO Composites

Surface properties	CuO	CuO-ZnO
Monolayer volume (cm^3^ (STP) g^–1^)	0.3240	1.2284
Specific surface area (m^2^ g^–1^)	1.41	5.35
Mean pore diameter (nm)	20.943	28.23
Total pore volume (cm^3^ g^–1^)	0.0074	0.0377

### 3.5 Nature of Surface Charge

To determine the isoelectric point (pH_PZC_) of CuO@ZnO nanoparticles, the pH drift method was followed where the pH of a NaCl solution was adjusted to acidic and basic conditions. The nanoparticles were then added, and it was kept in the dark for 48 h. After this period, the pH of each solution was measured and is recorded in Table 3. A graph of the initial pH versus final pH was plotted (Figure 6), revealing the pH_PZC_ for CuO@ZnO to be 7.8. This indicates that the composite acquires a positive charge when the pH of the dispersion medium is below 7.8 and a negative charge when above this value.

**Table 3:** Initial and Final pH Values Used to Determine the Point of Zero Charge (pHPZC) of CuO@ZnO Composites

Initial pH	Final pH
2	3.09
4	7.58
6	7.65
8	7.82
10	8.42
12	11.62

**Figure 6:**
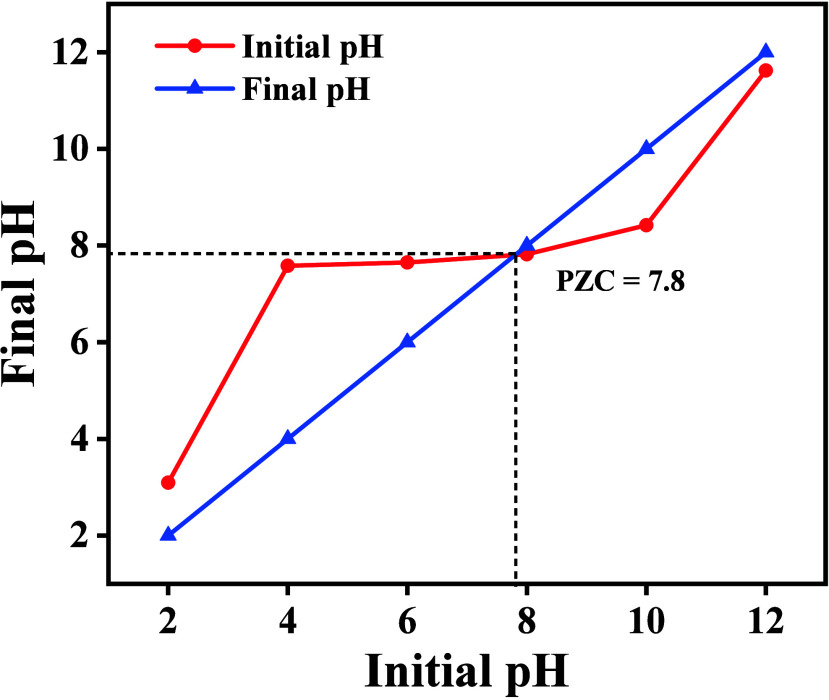
Initial versus final pH plot for C2 dispersions, used to determine the point of zero charge (pH_PZC_).

### 3.6 Photodegradation Studies

The synthesized semiconductor photocatalysts were evaluated with Rhodamine B dye as a model pollutant, which is generally found in most waste streams. The concentration of Rhodamine B was fixed to 10 μM, and the photocatalytic degradation test was conducted under natural sunlight between 11 am and 2 pm for nearly unchanging intensity. For the bare CuO, only 65% of the dye degraded in 90 min. Figure 7a shows the degradation of Rhodamine B dye under sunlight in the presence of the composite with different compositions at the natural pH of the dye. The degradation rate (Figure 7b) was found to be increased from 0.0096 min^–1^ to 0.0274 min^–1^ for the ZnO to CuO compositional ratio, respectively, from 1.5:1 to 2:1 (C1 to C2). With further increases in the ratio to 3:1 and 3.5:1 (C3 to C4), the rate, respectively, decreased to 0.0229 min^–1^ and 0.0187 min^–1^, indicating CuO to ZnO in the ratio 2:1 (C2) as the optimum composite.

**Figure 7:**
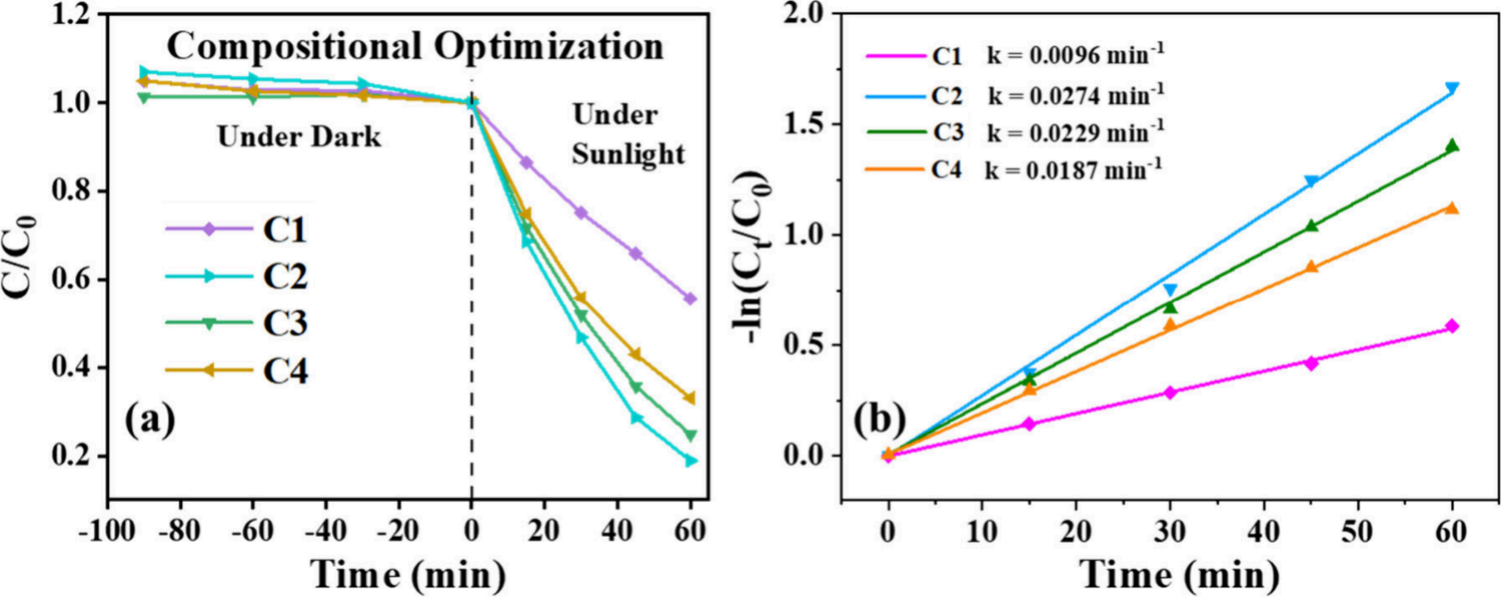
(a) Relative concentration of Rhodamine B over time during photocatalytic degradation by C1, C2, C3, and C4 composites under dark and sunlight conditions; (b) corresponding first-order kinetic plots.

#### 3.6.1 Optimization of pH of Dye Solution

The removal of RhB was also studied by varying pH, and the highest degradation was observed at the natural pH of the dye (Figure 8a,b). At higher pH levels, the catalyst dispersion lost stability, resulting in a lower surface area and consequently reduced degradation efficiency. Therefore, after optimization, the natural pH of the dye was used for all subsequent experiments.

**Figure 8:**
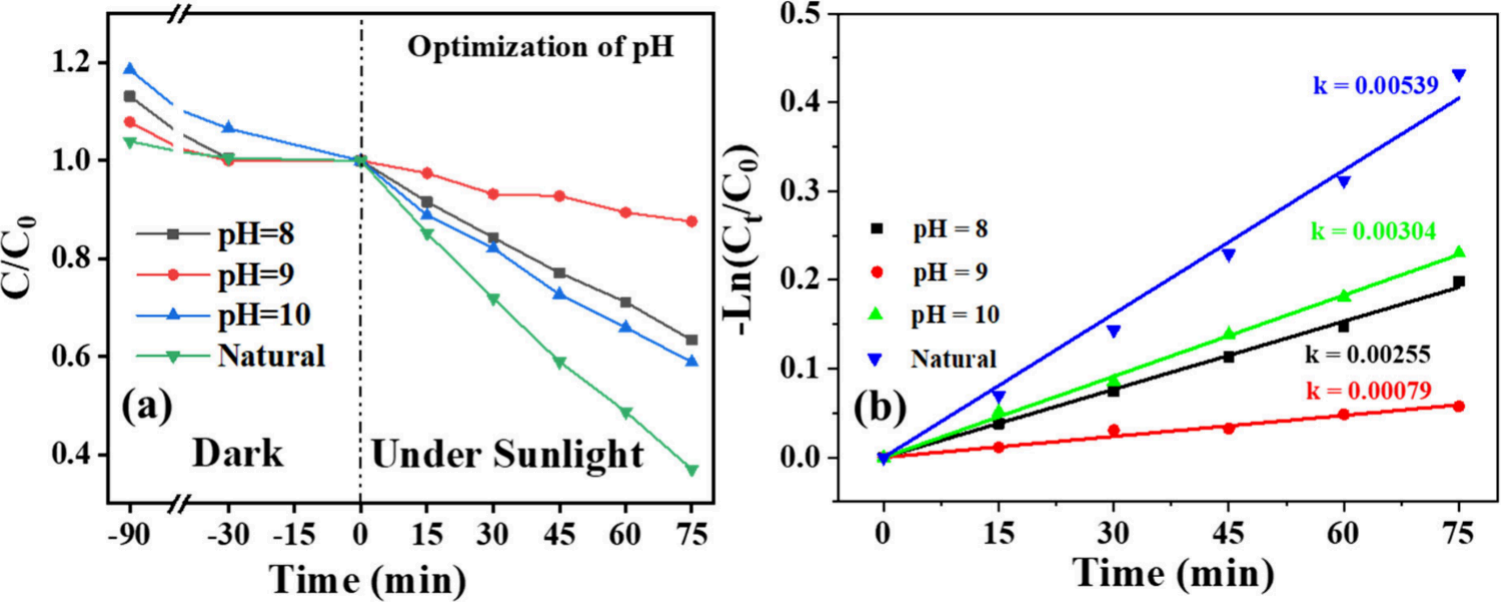
(a) Relative concentration of Rhodamine B over time during photocatalytic degradation by C2 composite under dark and sunlight at different pH values; (b) corresponding first-order kinetic plots.

#### 3.6.2 Optimization of the Photocatalyst Dosage

To optimize the catalyst loading, 15 mg, 20 mg, and 25 mg of the optimized composite C2 were dispersed in 50 mL of the dye solution and aged in the dark for 30 min before being exposed to sunlight (Figure 9a,b). The photocatalyst dosage of 20 mg in 50 mL resulted in the maximum degradation, indicating that a concentration of 400 mg/L is optimal. Typical spectral changes of RhB with time during photodegradation under sunlight in the presence of C2 are shown in Figure 10a,b.

**Figure 9:**
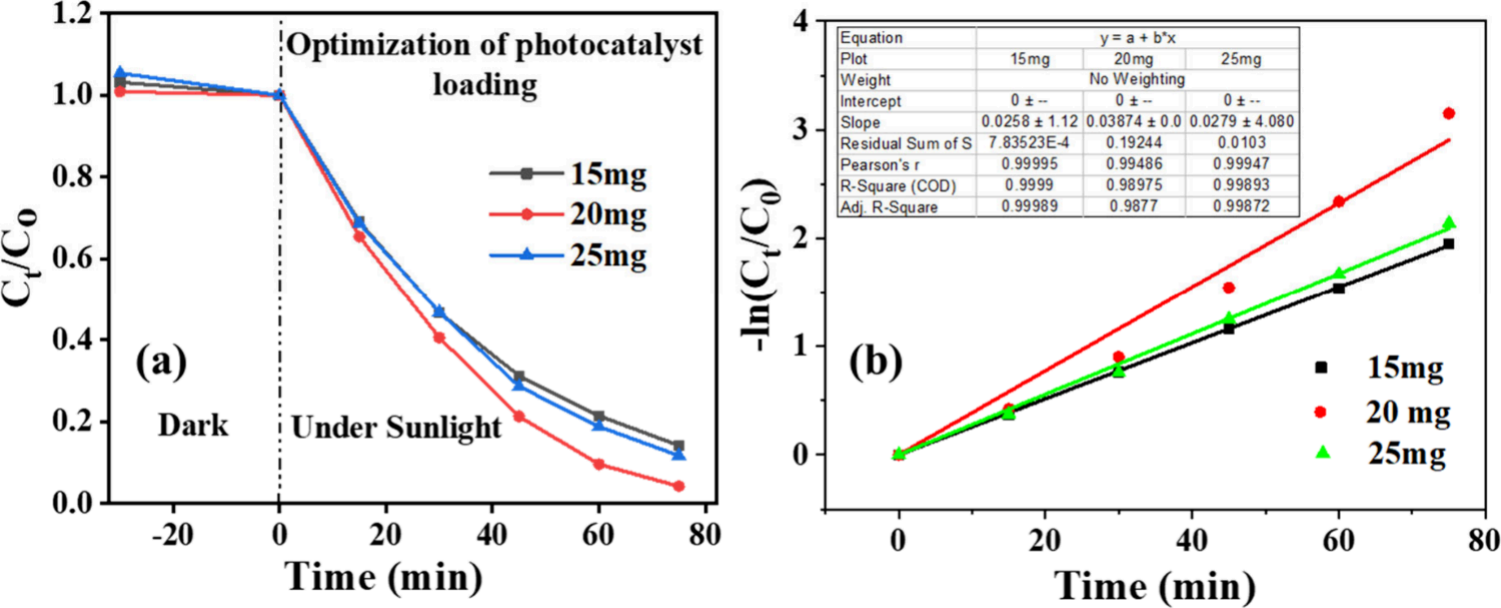
(a) Relative concentration of Rhodamine B over time during photocatalytic degradation by C2 composite at dosages of 15, 20, and 25 mg in 50 mL of dye solution; (b) corresponding first-order kinetic plots.

**Figure 10:**
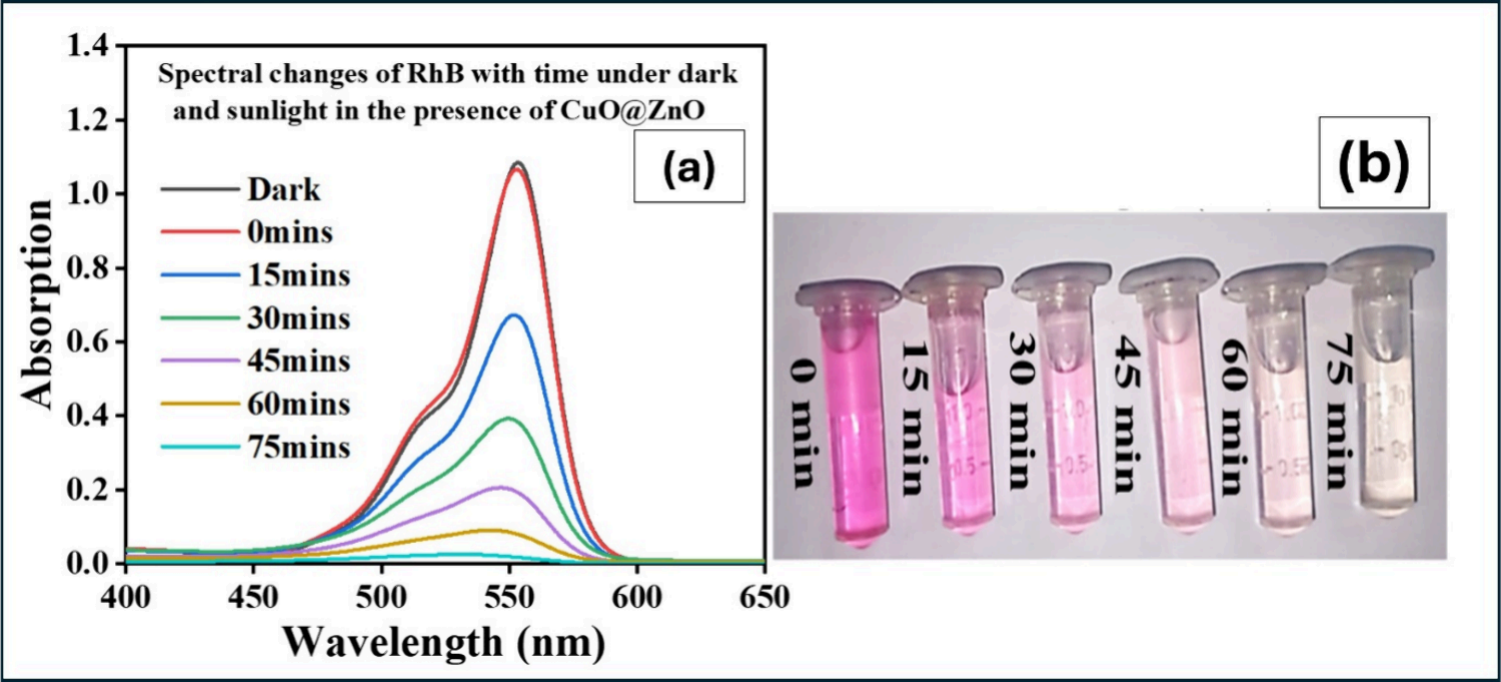
(a) UV–vis spectra of Rhodamine B under sunlight in the presence of C2 at different time intervals; (b) corresponding photographs of aliquots collected during degradation.

#### 3.6.3 Reusability Tests

Composite C2 was tested for its reusability by performing photocatalytic degradation with the repeated use of the same photocatalyst. As illustrated in Figure 11a, it was found that the photocatalyst offers consistent repeatable activity without any significant change for more than 5 cycles of reuse. The XRD patterns of C2 before and after the 5 cycles of photocatalysis indicated the composite was intact in its structural characteristics, as there were no changes in the patterns, proving that this catalyst can be used for multiple cycles without any regeneration of the catalyst (Figure 11b,c).

**Figure 11:**
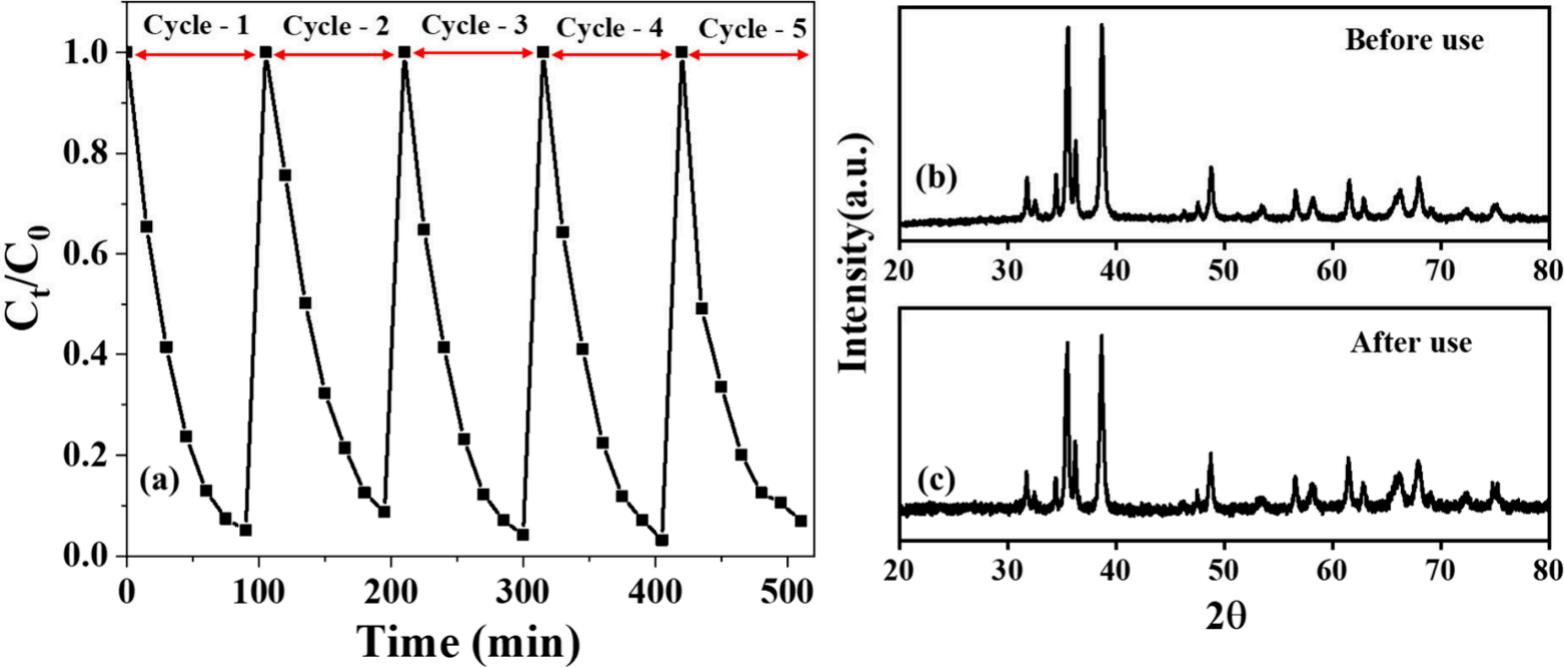
(a) Effect of C2 composite reuse on Rhodamine B degradation efficiency; (b, c) XRD patterns of C2 (b) before and (c) after five reuse cycles, demonstrating structural stability.

#### 3.6.4 Effect of Various Scavengers

To understand the degradation process, various scavenger tests were conducted to identify the reactive oxidative species involved in dye degradation (Figure 12). Hydroxyl radical scavengers, superoxide radical scavengers, and hole scavenger tests were performed. Tetra-butyl alcohol was used as a hydroxyl radical scavenger, resulting in 78% dye degradation. When p-benzoquinone was used as a superoxide radical scavenger, 46% of the dye was degraded. Finally, using potassium iodide as a hole scavenger, only 17% of the dye was degraded. These results indicate that holes play the major role in dye degradation directly, as shown in Figure 13.

**Figure 12:**
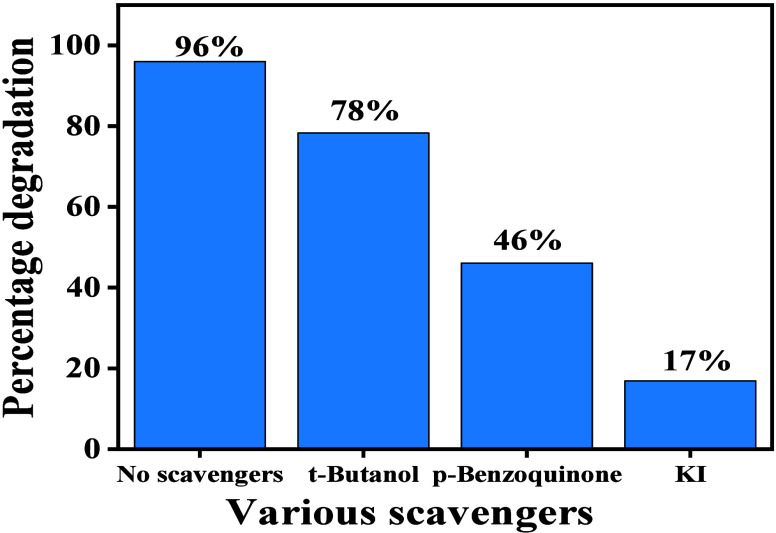
Percentage of Rhodamine B degradation in the presence of different reactive species scavengers, highlighting the roles of various reactive oxygen species in the photocatalytic process.

**Figure 13:**
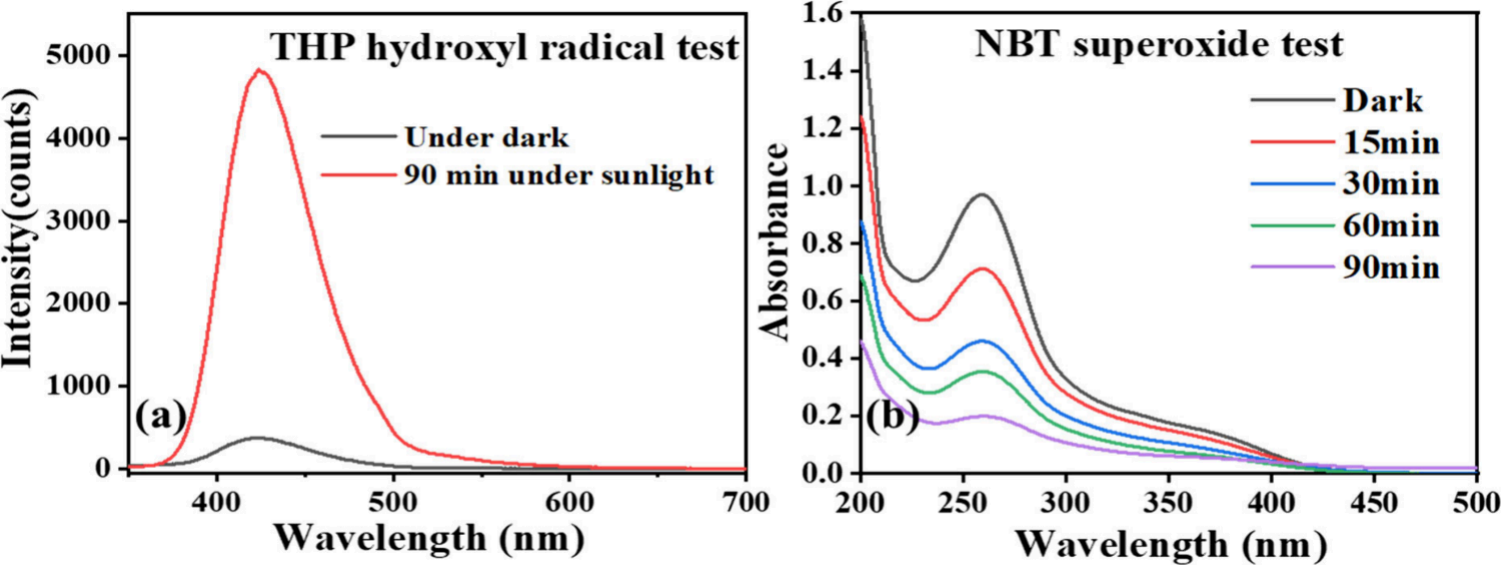
(a) Emission spectra of THP before and after sunlight exposure with C2; (b) Absorption spectra of NBT at various time intervals under sunlight in the presence of C2.

### 3.7 Mechanism of Photocatalytic Degradation of Rhodamine B in the Presence of C2

The photocatalytic mechanism over C2 is based on the type of heterojunction formed between CuO and ZnO (Figure 14). The heterojunction formation is decided by the Fermi levels/work function and band potentials of both the semiconductors of the composite.^12,37^ According to the published results, the work functions of CuO and ZnO are at ∼2.5 eV and ∼5.17 eV, respectively.^37,38^ The reported conduction band minima (CBM) for CuO and ZnO are −0.92 eV and −0.27 eV, respectively. Further, the valence band maxima for CuO and ZnO are respectively at 0.28 and 2.78 eV.^37,38^ Based on these parameters, it is inferred that ZnO acts as an oxidative catalyst and CuO as a reductive catalyst, and the higher work function of the oxidative catalyst (ZnO) than CuO implies the possible formation of S-scheme heterojunction.^37^ During the formation of the heterojunction, electrons move from the lower work function (high Fermi level) material (CuO) to the higher work function (low Fermi level) material (ZnO) until the Fermi levels equilibrated throughout the heterojunction.^17^ This migration of electrons resulted in a positive charge at CuO and a negative charge at ZnO that established the internal electric field directed from CuO to ZnO. When the C2 dispersion in Rhodamine B solution is exposed to sunlight, electron–hole pairs are generated in ZnO and CuO. The electrons in the conduction band of ZnO neutralized the holes in the valence band of CuO, leaving the accumulation of electrons and holes on the surface of CuO. These accumulated electrons and holes in two different materials initiated the oxidative and reductive reactions, respectively, that resulted in the generation of reactive oxygen species. The holes in the conduction band of ZnO oxidize water or hydroxide ions to produce hydroxyl radicals. Similarly, the accumulated electrons on the surface of CuO reduced oxygen molecules to produce superoxide radicals, which subsequently generated hydroxyl radicals. The generation of hydroxyl and superoxide radicals was evident by the confirmatory tests, as discussed in section 3.6.4 (Figure 13). These generated reactive species actively participated in the degradation of Rhodamine B.

**Figure 14:**
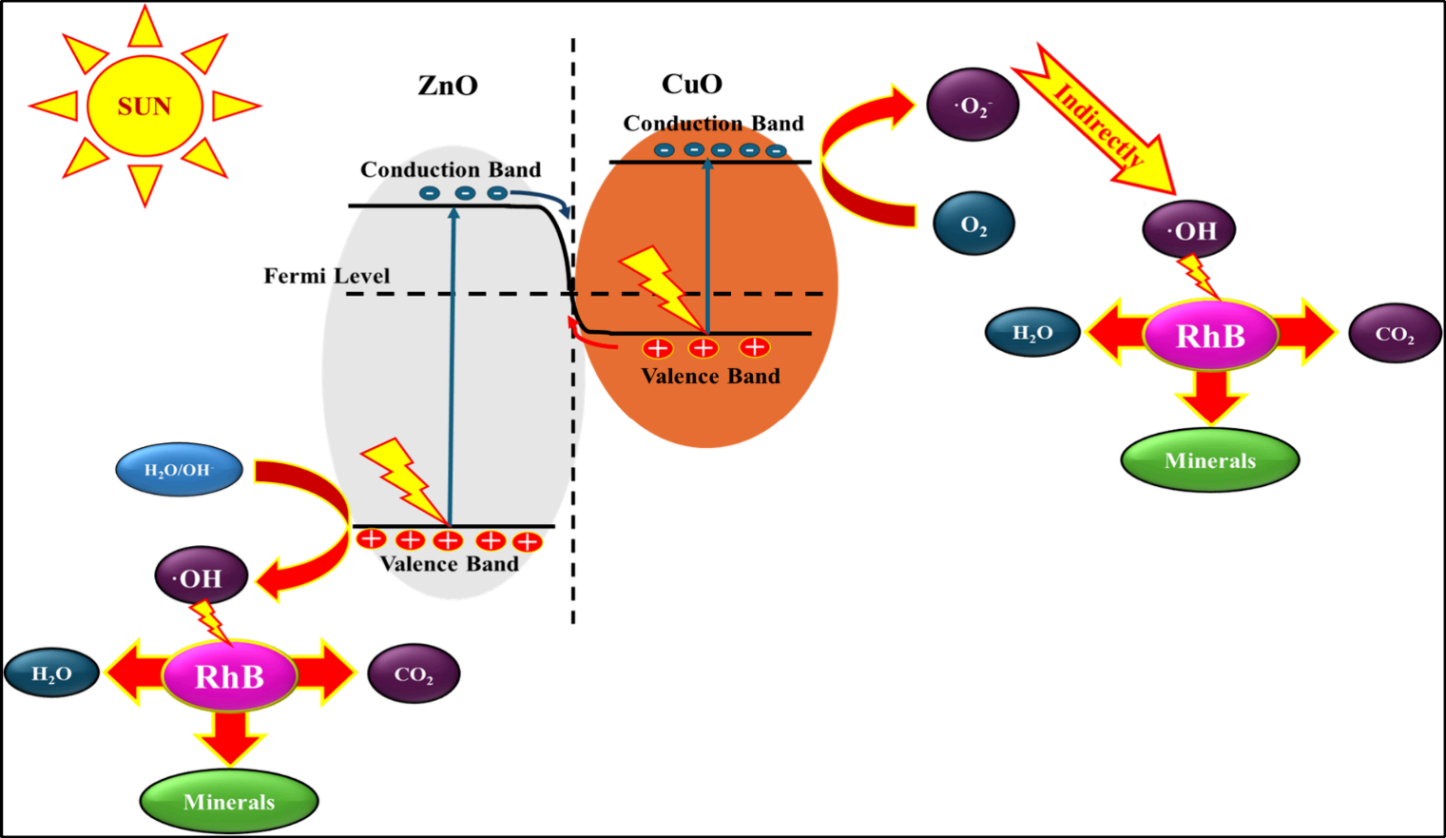
Proposed mechanism of Rhodamine B degradation over the C2 composite under sunlight, illustrating charge separation and reactive species generation.

In summary, the outstanding photocatalytic performance of the CuO/ZnO composite is mainly due to the formation of a S-scheme heterojunction between the two semiconductors: CuO and ZnO. In this arrangement, CuO acts as a p-type semiconductor with a narrow bandgap, while ZnO serves as an n-type semiconductor with a wider bandgap. This intimate interface creates a unique charge transfer pathway upon exposure to solar irradiation. When the composite is exposed to sunlight, both CuO and ZnO absorb photons and generate electron–hole pairs. The S-scheme heterojunction facilitates the selective migration of photogenerated electrons from the conduction band of ZnO and holes from the valence band of CuO, while the less energetic charge carriers (electrons in CuO and holes in ZnO) recombine at the interface. This spatial separation preserves the electrons in ZnO and holes in CuO, which possess strong redox potentials: electrons in ZnO efficiently reduce oxygen to generate reactive oxygen species (ROS), while holes in CuO oxidize water or organic pollutants. This efficient charge separation strongly suppresses electron–hole recombination, prolongs the lifetime of charge carriers, and enhances the generation of ROS, thereby significantly improving the photocatalytic degradation of organic pollutants under full-spectrum sunlight. The S-scheme mechanism thus synergistically combines the advantages of both semiconductors, leading to the superior activity, stability, and reusability of the photocatalyst.

## 4 Conclusions

This work demonstrates the successful design and synthesis of biomimetic porous CuO–ZnO microspheres with enhanced photocatalytic performance for dye degradation under solar irradiation. By combining microwave-assisted hydrothermal synthesis with heterojunction engineering, we achieved significant improvements in active site availability and charge carrier separation, which contributed to the excellent photocatalytic efficiency and stability. The identification of key reactive species and the durability of the photocatalyst over repeated cycles underscore its practical applicability. These findings advance the development of cost-effective, eco-friendly photocatalysts for sustainable wastewater treatment, addressing critical environmental challenges related to water pollution and scarcity. Thus, this work provides an important contribution toward scalable, solar-driven technologies for environmental remediation.

## §

Department of Inorganic Chemistry, Faculty of Natural Sciences, Comenius University Bratislava, Ilkovikova 6, Mlynska dolina, 842 15 Bratislava, Slovak Republic
